# Illinois State Medical Society

**Published:** 1879-07

**Authors:** N. S. Davis

**Affiliations:** Per. Secretary


### ARTICLE X.

ILLINOIS STATE MEDICAL SOCIETY, Proceedings of the Twenty-Ninth Annual Meeting, held in Lincoln, on the 20th and 21st days of May, 1879.

The members of the Society assembled in the Opera House, and were called to order by the President, Dr. E. P. Cook, of Mendota, at 11 o'clock A. M. Prayer was offered by the Rev. George Stevens, of Lincoln; after which, T. T. Beach, Esq., in behalf of the Committee of Arrangements and citizens of Lincoln, welcomed the members of the Society to the hospitalities of the city in an appropriate and eloquent address. He complimented them as the conservators of the public health; and spoke of their annual gatherings as admirably calculated to promote the education, advance the science and to cultivate the social intercourse and friendship of the profession. He alluded to the ancient origin of the profession, and to the wonderful progress made in its science and usefulness in modern times. He spoke of the discoveries of Harvey, Jenner and others, and the opposition they had met from their contemporaries, and their ultimate triumph. He commended the faithfulness of the profession in the discharge of the most arduous duties at all times, and under all circumstances, to rich and poor alike. He did not think there was any class of community so ready to extend aid to the poor. He approved the laws establishing a State Board of Health and regulating the practice of medicine, saying that they had already done much good and ought to be sustained both by the profession and the community. He closed by again cordially welcoming the society to the hospitality of the profession and citizens of Lincoln.

The President called upon Dr. E. Ingals, of Chicago, to respond to the address. He said on taking the stand, that some of his colleagues from Chicago must have been playing a joke on him by inducing the President to call him up in the midst of so many so much more able to respond in fitting terms to the eloquent and cordial welcome to which we had just listened. We had come together for a brief respite from the round of daily toil, to advance our knowledge, to re-kindle our social feelings and to strengthen the ties of friendship, and in behalf of the society he fully reciprocated the kindly sentiments that had been so happily expressed. He did so with all the more pleasure because they had been uttered by a distinguished member of the legal profession. A profession which had, indeed, done much for the doctors, especially while having them on the stand as expert witnesses.

Dr. R. P. Wilson, Chairman of the Committee of Arrangements, presented a programme, recommending that the morning meetings commence at 9½ A. M., and the afternoon meetings at 2½ P. M., and extending invitations to attend an exhibition of the pupils of the Asylum for Feeble Minded Children, on Tuesday evening; a puplic address by Dr. N. S. Davis, on Wednesday evening, and at a later hour the same evening, a reception by Genl. and Mrs. Latham. The programme and invitations were accepted by a vote of the society.

The Secretary read a letter from Dr. E. Swisher, asking permission to withdraw from membership in the society, on account of having removed from the State. The request was granted, and on motion of Dr. T. D. Fitch, Dr. Swisher was unanimously elected an honorary member of the society.

The following were introduced by the Committee of Arrangements and took their seats as members by invitation: Drs. J. W. Downey, of Pekin; B. F. McMennany, of Bethany; A. C. Reno, of Albion; O. C. Reynolds, of Illinois; P. H. Oyler, of Mount Pulaski; A. H. Mersenback, of Illinois; Hosea Davis, of Littleton; Brooks R. Hamilton, of Nauvoo; J. J. Starkey, of Waynesville; A. D. Taylor, of Williamsville.

The list of standing and special committees was called, and special hours were designated when the report from each should be read. Volunteer papers were also called for and assigned to particular hours for being read and discussed.

The President, Dr. E. P. Cook, of Mendota, then delivered the annual address, which was listened to with great interest and attention. After its conclusion, the thanks of the society were tendered to the speaker. The recommendations were referred to a special committee, consisting of Drs. David Prince, G. W. Nesbitt, and H. Z. Gill, with instructions to report at this or the next annual meeting, as they might determine; and a copy of the address was requested for publication in the transactions of the society. The society then adjourned until 2½ P. M.

AFTERNOON SESSION.

At 2½ P. M., the members were called to order by the President, Dr. E. P. Cook, of Mendota.

Dr. D. Prince, of Jacksonville, read a short and interesting paper on the "Sanitation of Small Cities," which was received and referred to the Committee of Publication.

Dr. J. E. Owens, of Chicago, presented and read the report from the standing Committee on Surgery. It was listened to with interest and close attention, and was followed by a profitable discussion, in which Drs. E. Andrews, J. A. Walker, D. Prince, and J. H. Hulbert participated. (See remarks following the report.)

On motion of Dr. Prince the report was referred to the Committee of Publication.

Dr. Truesdell, of Rock Island, read an interesting paper on fractures of the femur, which was followed by remarks from Drs. D. Prince and H. Z. Gill. (See notes following the paper.)

On motion of Dr. Prince the paper was referred to the Committee of Publication.

Dr. J. H. Hollister called attention to the rule that all papers of more than fifteen pages of foolscap manuscript should be presented and read by abstract.

Dr. C. C. Hunt, of Dixon, presented the report of the standing Committee on Obstetrics, and read an abstract of the same.

On motion of Dr. E. Ingals the report was received and referred to the Committee of Publication.

Dr. S. J. Jones, of Chicago, presented and read the report of the Committee on Ophthalmology and Otology, which, on motion of Dr. E. Ingals, was received and referred to the Committee on Publication.

Dr. H. M. Lyman, of Chicago, read a paper on State Medicine, which was discussed to a limited extent by Drs. Hollister, Prince, Ingals, and Gill; and on motion of Dr. J. H. Hollister, was laid upon the table, to be taken up again at the pleasure of the society.

The meeting then adjourned until 9 A. M., the following morning.

WEDNESDAY MORNING, 9 A. M.

The members were called to order by the President, Dr. E. P. Cook, who announced the first order of business to be the appointment of the nominating committee.

A recess of ten minutes was taken to enable the members from each county represented to select one of their number to act on the committee.

On being called to order, the secretary read the names of those selected, as follows:

*Committee on Nominations.*—Drs. D. M. Slemmons, Woodford county; F. B. Waller, Fayette county; G. W. Albin, Cumberland county; A. C. Corr, Macoupin county; O. C. Reynolds, Christian county; A. C. Rankin, Iriquois county; W. E. Gilliland, Adams county; R. E. McVey, Morgan county; E. L. Herriott, Calhoun county; W. R. McKinzie, Randolph county; M. W. Walton, Stephenson county; C. Chenoweth, Macon county; C. C. Hunt, Lee county; H. Kelso, Ford county; A. T. Darrah, Champaign county; J. W. Newcomer, Menard county; G. W. Nesbitt, DeKalb county; T. M. Rogers, Wayne county; J. M. Armstrong, Madison county; H. Hatch, Pike county; W. J. Moore, Vermillion county; J. W. Fink, Montgomery county; H. Z. Gill, Jersey county; C. C. Allen, Brown county; B. M. Griffith, Sangamon county; W. West, St. Clair county; J. A. Walker, Mason county; J. J. Starkey, De Witt county; O. B. Hill, Peoria county; E. P. Cook, La Salle county; T. D. Fitch, Cook county; R. W. Crothers, Tazewell county; W. Hill, McLean county; N. S. Reed, Cass county; W. T. Kirk, Logan county; T. J. Maxwell, Henderson county; C. Truesdell, Rock Island county.

Dr. G. Wheeler Jones, of Danville, then presented and read the report of the Committee on Practical Medicine, which was listened to with marked attention, and referred to the Committee of Publication.

Chairman of the Committee of Arrangements read an invitation from Mrs. Col. Latham tendering the members of the State Medical Society a reception at her residence on Wednesday evening. On motion the invitation was accepted.

Dr. C. H. Norred read a paper on the Uses of Chloral and the Bromides in Obstetric Practice.

The paper was accepted and referred to the Committe of Publication.

Dr. E. Ingals, Chairman of the special Committee on Medical Education, read a report that was listened to with much interest.

The following resolutions were appended to the report:

*Resolved*, That the Illinois State Medical Society requests of all regular medical colleges, that they institute preliminary examinations for students who apply for admission to their classes, and only admit such as have, at least, a thorough English education.

*Resolved*, That the annual sessions of lectures by the regular faculties should not be of shorter duration than six months.

*Resolved*, That all students should be required to study medicine five years and attend three full annual sessions of lectures before they are admitted to examination for the degree of Doctor of Medicine.

On motion the report was accepted and referred to the Committee of Publication; and the resolutions were adopted, the first two unanimously, and the third with but few negative votes.

Short papers were presented and read by Drs. E. Andrews and E. L. Holmes, and referred to the Committee of Publication.

The society then adjourned until 2½ P. M.

### AFTERNOON SESSION.

At 2:30 p.m. the Society was called to order by the President.

Dr. T. D. Fitch, of Chicago, read the report of the Committee on Gynæcology, which was received and referred to the Committee of Publication, without discussion.

Dr. T. F. Worrell, of Bloomington, chairman of the Committee on Necrology, stated that he had a report nearly completed, but it had been accidentally left at home. On motion, he was requested to complete his report and transmit it to the Committee of Publication.

Dr. Sarah Hacket Stevenson, of Chicago, read a paper on disorders of the Sympathetic Nervous System, illustrated by cases. The paper was discussed by Drs. Jewell and Stevenson, after which it was referred to the Committee of Publication.

Dr. H. Z. Gill, of Jerseyville, read his report as Special Committee on Croup; and also a short paper as supplementary to the report of the Committee on Surgery.

The report on croup was illustrated by two finely executed plates, showing the anatomy of the parts, which the writer requested permission to retain.

On motion of Dr. N. S. Davis, his request was granted, and both the report and paper were referred to the Committee of Publication.

Dr. Eli Brower, of Olney, chairman of the Special Committee to whom had been referred the communication on the subject of remuneration to medical witnesses in courts of justice, etc., at the last annual meeting, sent a full and interesting report, which was read by the Secretary and referred to the Committee of Publication.

Dr. J. H. Hollister, of Chicago, presented his annual report as treasurer of the Society. It had been duly audited, and was referred for publication.

Dr. B. M. Griffith, of Springfield, chairman of the Nominating Committee, made the following report:

*To the President and Members of the Illinois State Medical Society,*

GENTLEMEN:—Your Committee on Nominations met at the Lincoln House parlors at 12 m. to-day, all present except the President and Dr. N. S. Read. They beg leave to submit as the results of their deliberations, the following report, viz.:

For President, Ephraim Ingals, Chicago; 1st Vice-President, George Wheeler Jones, Danville; 2d Vice-President, C. C. Hunt, Dixon; Treasurer, John H. Hollister, Chicago; Assistant Secretary, Washington West, Belleville. Place of meeting, Belleville.

*Committee of Arrangements.*—T. B. Moore, Belleville; Washington West, Belleville; T. M. Armstrong, Madison county; Wm. R. McKenzie, Randolph county; H. Z. Gill, Jerseyville.

*Committee on Practical Medicine.*—W. S. Caldwell, Warren; F. B. Haller, Vandalia; I. S. Whitmire, Metamora.

*Committee on Surgery.*—W. Hill, Bloomington; A. C. Rankin, Loda; J. G. Harvey, Grove city.

*Committee on Obstetrics.*—G. W. Nesbitt, Sycamore; E. L. Harriott, Grafton; I. W. Fink, Hillsboro.

*Committee on Gynæcology.*—T. Davis Fitch, Chicago; J. Y. Campbell, Paxton; L. H. Corr, Carlinville.

*Committee on Ophthalmology.*—W. T. Montgomery, Chicago; J. M. Everett, Dixon; J. P. Johnson, Peoria.

*Committee on Drugs and Medicines.*—C. B. Johnson, Champaign; Jehu Little, Bloomington; Thos. Whitten, Irving.

*Committee on Necrology.*—T. F. Worrell, Bloomington; G. W. Albin, Neoga; E. Ingals, Chicago.

*Vacancies in Judicial Council.*—F. B. Haller, Vandalia; E. Ingals, Chicago; M. W. Walton, Ridott.

*Committee on State Register.*—E. P. Cook, Mendota; F. L. Matthews, Springfield; D. S. Boothe, Sparta.

*Special Committees.*—Croup: H. Z. Gill, Jerseyville; Diseases of Children: Chas. W. Earle, Chicago; M. W. Watton, Ridott.

*Pelvic Cellulitis* (Parametritis).—P. L. Dieffenbacher, Havana; Pneumonia: J. Maclay Armstrong; Antiseptic Surgery: Edwin Powell, Chicago; Abnormal Thermal Conditions in Diseases and the Means of Controlling them: J. H. Hollister, Chicago.

The following resolution was unanimously adopted by your committee, viz.:

*Resolved*, That hereafter the Illinois State Medical Society shall meet in Chicago every third year.

B. M. GRIFFITH, *Chairman.*

T. D. FITCH, *Secretary.*

The report was received, and Dr. J. H. Hollister moved to substitute Chicago for Belleville, as the place for the next annual meeting.

After some remarks from Drs. Waller, Rauch, Davis and Jewell, the motion was rejected.

The whole report except the appended resolution was then adopted.

A motion was made to lay the resolution on the table, which was carried.

The Secretary presented a communication from the Centennial Medical Society, concerning Weak, Impure and Adulterated Drugs, which was read and referred to the standing Committee on Drugs and Medicines.

Dr. W. West offered the following, which was unanimously adopted:

*Resolved*, That the Illinois State Medical Society regards with satisfaction and hearty approval, the efforts of Hon. Wm. R. Morrison to repeal the onerous duty on quinine.

Dr. D. Prince, of Jacksonville, presented the following report, which was adopted:

*Resolved*, As the sense of the Illinois State Medical Society that the forms of law adopted for estabishing a question of crime, are unsuited to the determination of a question of insanity, on account of the exposure to public curiosity and the supposed disgrace attending a trial by jury; and that this mode of procedure should be reserved for the cases in which it is requested by the parties who are suspected to be insane, or by the friends of such parties, and who are desirous of establishing by this means the mental soundness of the person in question.

*Resolved*, That the bill now pending in the Legislature of the State of Illinois, entitled An Act to revise the Law in Relation to Commitment and Detention of Lunatics, meets with the hearty approval of the Illinois State Medical Society. And that in the interests of humanity and for the credit of our own State, this Society respectfully prays that the Legislature now in session will speedily adopt the provisions of said bill as the law of our State.

DAVID PRINCE,<br>

H. Z. GILL, } *Committee.*<br>

G. W. NESBITT,

Dr. R. E. Starkweather moved that the secretary be directed to cause the foregoing resolutions to be presented to the Legislature without delay, which was adopted.

On motion the society adjourned until after the public address in the evening.

### EVENING SESSION.

At 8 o'clock p. m., the hall in which the meetings had been held was filled by a reputable assembly of citizens, and Dr. N. S. Davis, of Chicago, delivered an address on the Nature of Medical Science and Art, and their Relations to the Important Interests of Society.

At the close of the address, Dr. E. P. Cook, President of the Society, took the chair and resumed the order of business.

Dr. G. W. Nesbitt, of Sycamore, offered a resolution favoring a law allowing physicians compensation for making returns of certificates of deaths, births, etc.

After some remarks in opposition by Drs. Davis, of Chicago, and Jones, of Jacksonville, and in favor by Dr. Lyman, of Chicago, the resolution was laid on the table.

On motion of Dr. T. D. Fitch, the Committee of Publication was directed to furnish all the medical periodicals, copies of the transactions of the Society, and if necessary, bind a sufficient number in paper for that purpose.

Dr. G. W. Nesbitt, of Sycamore, offered the following resolutions, which were adopted:

WHEREAS, In the opinion of the Illinois State Medical Society, many diseases are generated and propagated by the crowded and unventilated condition of our public school houses; therefore be it

*Resolved*, That a committee of three be appointed by the chairman of this Society, to memorialize the Legislature of the State of Illinois, at its next session, to enact a law establishing a board of commissioners of hygiene, consisting of the County Superintendent of Schools, County Surgeon, and one physician, in each county in this State, whose duty it shall be to inspect all public school houses, and see that they are supplied with proper means of ventilation, and are of sufficient size to accommodate the number of pupils in attendance; to inspect all proposed sites and plans of new school houses, and approve or reject the same; and to adopt such measures for the preservation of health in our public schools as from time to time they may think necessary and proper. Provided, that no measures so adopted shall conflict with the rights or duties of any local board or officer already established.

*Resolved*, That the committee so appointed to memorialize the Legislature be instructed to prepare a bill embodying the substance of the foregoing resolution, and present the same to this Society at its next session, for their approval, before introducing the same to the Legislature of the State.

The President appointed as a committee under the above resolution Drs. G. W. Nesbitt, of Sycamore, H. M. Lyman, and J. N. Rauch, of Chicago.

Dr. N. S. Davis offered the following resolutions, which were unanimously adopted:

*Resolved*, That the thanks of this Society are most cordially tendered to the Committee of Arrangements for the faithful and judicious manner in which they have provided accommodations for our present meeting.

*Resolved*, That the thanks of the Society are hereby tendered to Dr. C. L. Wilbur and the officers of the State institutions for the education and care of feeble minded children, for the very interesting exhibition of progress and excellence in management the members of the society had the pleasure of witnessing on Tuesday evening.

*Resolved*, That our thanks are also tendered to the profession and citizens of Lincoln generally, and to Col. and Mrs. Latham in particular, for the hospitality and elegant and enjoyable reception at the residence of the latter.

The following were appointed delegates to the American Medical Association, and the State Medical Societies: G. W. Frank, of Hillston; E. P. Cook, of Mendota; G. W. Nesbitt, of Sycamore; C. H. Norred, of Lincoln; Wm. Hill, of Bloomington; W. J. Moore, and G. W. Jones, of Danville; W. M. Chambers, of Charleston; C. C. Hunt, of Dixon; W. G. Cochran, of Fair City; C. Goodbrake, of Clinton; S. W. Stevenson, and T. D. Fitch, of Chicago; G. W. Albin, of Neoga; G. W. Newcomer, of Petersburg; E. Ingals, of Chicago; T. G. Maxwell, of Biggsville; E. L. Herriott, of Grafton; Saml. Milcher, of Cass Point; J. H. Hollister, of Chicago; Lucede H. Caxar, of Carlinville; G. F. Worrell, of Bloomington; D. Paine, of Jacksonville; R. L. Rea, of Chicago; L. L. Leeds, of Lincoln; R. E. Starkweather, Chicago; N. Wright, of Chatham; C. T. Wilbur, of Lincoln; E. Andrews, of Chicago; J. D. Bennett, of Assumption; J. R. Jones, of Lemoyle; E. L. Holmes, of Chicago.

*Missouri State Medical Society.*—Drs. E. L. Herriott, Grafton, and J. S. Williams, Otterville.

*Indiana State Medical Society.*—Drs. E. T. Pritchard, Georgetown, and S. H. Berry, Chicago.

*Michigan State Medical Society.*—Drs. E. Andrews, and S. J. Jones, Chicago.

*Kentucky State Medical Society.*—Dr. Wm. T. Kirk, Alton.

*Wisconsin State Medical Society.*—Drs. T. D. Fitch, J. S. Jewett, and R. L. Rea.

*Iowa State Medical Society.*—Dr. Thos. Gault, Ridott.

The business of the Society having been completed, the President, Dr. E. P. Cook, made some appropriate remarks, congratulating the members on the pleasantness and success of the session, and introduced the President elect, Dr. E. Ingals, of Chicago, who was received with hearty cheers. The Society then adjourned *sine die*.

N. S. DAVIS. *Per. Secretary.*

---

ARTICLE XI.

