# Illinois State Medical Society

**Published:** 1879-08

**Authors:** 


### Editorial.

### ARTICLE XVI.

### ILLINOIS STATE MEDICAL SOCIETY.

In compliance with the request of one of the editors of this journal, the Secretary of the State Medical Society furnished the full official record of the proceedings of the recent annual meeting for the July number. The manuscript was contributed rather late in the month, and by some mistake or oversight, it was put to press without allowing the Secretary to see a copy of the proof sheets. The consequence is a lamentable number of typographical errors, mostly in the names of persons and places. In the first of the resolutions offered by Dr. Nesbitt, printed on page 86, instead of " County *Surgeon* " it should be County *Surveyor*. The number of papers read before the State Society during its recent meeting was large, and they were well written; and with the record of proceedings will make a volume of transactions containing between two and three hundred pages. Besides the reading and discussion of reports and papers of value relating to the science and art of medicine, the attention of the society was given to several other topics of general interest. Both in the address of the president and the report of a special committee, the sentiments of the society were strongly and unanimously expressed in favor of amending the present State law in relation to the mode of determining questions of insanity with reference to restraining the insane, or committing them to the care of asylums. It will be seen by reference to the proceedings that the report of the committee on this subject was brief and judicious. That report, accompanied by the endorsement of the society, was immediately presented to the legislature, by Dr.

Nesbitt, listened to with attention, and referred to the committee having the bill for amending the law in charge. The legislature, however, subsequently adjourned without passing the bill, thereby still leaving upon the statutes of our State the arbitrary, and in some respects cruel, provision that every person charged with insanity or mental unsoundness, must be brought into open court, confronted with witnesses and tried by a jury. Another subject worthy of attention, was embodied in the preamble and resolutions offered by Dr. G. W. Nesbitt, of Sycamore, asking for the framing and enactment of a law establishing in each county in the State, a "board of commissioners of hygiene," to consist of the County Superintendent of Schools, County Surveyor and one physician, whose duty it should be to exercise a superintendence over the construction and sanitary condition of all public school buildings and locations. A committee consisting of Drs. G. W. Nesbitt, H. M. Lyman and J. H. Rauch, was appointed to draft a law and report at the next annual meeting of the society. The object sought to be obtained is one of great importance, and should receive the earnest practical attention of every intelligent physician, whether he is appointed on a commission of hygiene or not. And yet we think every proposition to increase the number of municipal, county or State officers or commissioners, should be viewed with some distrust.

There is too great a tendency to relieve the people of responsibility by committing their duties to commissioners or some kind of public officers, and yet all experience shows that, in nine cases out of ten, such official positions are filled with mere political partisans, who are more anxious for the success of the political party than for the health and welfare of any part of the community. In matters of hygiene, especially in regard to the management of children and schools, every physician should consider himself responsible for the proper education and instruction of every family to which he has access, and for an active interest in the sanitary condition of every school building in his neighborhood. In other words, every educated physician should be an active conservator of the health of the community in which he lives, and not a mere *prescriber* for the sick.

